# A New Reactor Concept for Single-Chamber Microbial Fuel Cells and Possible Anti-Fouling Strategies for Long-Term Operation

**DOI:** 10.3390/microorganisms10122421

**Published:** 2022-12-07

**Authors:** Dennis R. Haupt, Laura Landwehr, René Schumann, Lena Hahn, Mohammad Issa, Can Coskun, Ulrich Kunz, Michael Sievers

**Affiliations:** 1 CUTEC Clausthal Research Center for Environmental Technologies, Clausthal University of Technology, 38678 Clausthal-Zellerfeld, Germany; 2 Institute of Chemical and Electrochemical Process Engineering, Clausthal University of Technology, 38678 Clausthal-Zellerfeld, Germany

**Keywords:** microbial fuel cell, gas diffusion electrode, power generation, anti-fouling, wastewater treatment

## Abstract

Microbial fuel cells are a promising technology for future wastewater treatment, as it allows cleaning and power generation simultaneously. The bottleneck of microbial fuel cells is often its cathodes because they determine the power output. Gas diffusion electrodes might overcome this bottleneck due to their low production costs and high oxygen reduction rates. However, biofilm formation on the gas diffusion electrodes reduces their performance over time. In this work, a new reactor design of the microbial fuel cell using rotating gas diffusion electrodes is presented. The biofilm growth on the electrode during operation was observed and its effect on the performance of the microbial fuel cell was examined. In addition, different antifouling strategies were investigated over a period of 80 days. It was found that already after 7 days of operation a complete biofilm had grown on an untreated gas diffusion electrode. However, this does not seem to affect the performance of the cells in the beginning. Differences in the performance of the reactors with and without an antifouling strategy only become apparent from day 15 onwards. The use of UV radiation and antibacterial membranes leads to the best results with maximum power densities of approx. 200 mW m^−2^ while the untreated microbial fuel cell only achieves a maximum power density of approx. 20 mW m^−2^.

## 1. Introduction

Microbial fuel cells (MFCs) are a promising technology for simultaneous wastewater treatment and power generation [1]. From the early 20th century [2] until today, uninterrupted research activities can be registered. Despite several thousands (>18,000) of publications even in the year 2020 (Google scholar, keyword microbial fuel cell), there is no commercial breakthrough, yet [3]. MFCs are still in the stadium of basic research. The reasons for that are manifold, starting from high investment costs, high operational costs [4], still low power output [5], and the use of toxic materials such as potassium ferricyanide [6]. Several research groups identified especially the cathode as the bottleneck, which means that the cathode determines the power generation and the operation (time) of the MFC [7]. For that reason, new cathodes have to be developed, which deliver high and stable electrical power outputs [8].

Gas diffusion electrodes (GDEs) are an encouraging technology to overcome that bottleneck as they can be made of unharmful materials such as carbon and additional catalysts for oxygen reduction reaction (ORR) and they are also able to deliver a high electric output [9]. However, GDEs are prone to fouling, which affects long-term stability and leads to a decrease in cell performance over time. Therefore, more and more antifouling methods are being researched.

Table 1 shows an overview of selected MFCs with antifouling investigations and their power densities.

Anti-fouling strategies can be categorized into two groups: in situ strategies and non-in situ strategies. In situ means that the anti-fouling strategy can be operated directly in the reactor without any GDE-disassembling. This minimizes operational costs and prevents operational outages, especially regarding a future technical operation. For that reason, five possible in situ strategies were selected and investigated: UV radiation (1), coating with copper compound (2), polarity reversal (3), rotation speed adjustment (4) and membrane coating (5). All approaches were carried out once to examine their feasibility and effectiveness. The details of each strategy are as follows:

(1) UV light is known for its antibacterial effect. The wavelength of approx. 240–290 nm affects the RNA and DNA of organisms and this leads to inactivation [25]. UV light disinfection is state of the art in drinking water disinfection [26]. When using UV rays against fouling, it has been shown that higher intensity improves the protection against fouling [27]. The required effective dose varies depending on the type of bacteria, mold spores and algae [28]. In addition, a higher exposure time also improves the antifouling effect [29].
(2) Copper is known for its biocidal effect on a wide range of microbials [30] and application spectrums, e.g., hospitals [31]. Therefore, copper or copper compounds are a promising method to prevent bacterial growth on the surface of the GDE.
(3) Polarity reversal is known in electrochemical systems to prevent the cathode from scaling. During polarity reversal, the cathode becomes the anode and oxidation process occurs on the former cathode [32]. This technique has to be adapted for use in MFCs.
(4) Mechanical ablation by increasing the shearing force is another option for removing parts of the biofilm. Conventional ablation by using blades or wipers was not possible, as determined in prior experiments. Any direct contact with blades or wipers destroys the surface of the GDE, whereby gaps and consequently electrolyte breakthroughs occur. For that reason, shear forces should remove the biofilm of the GDE. It is known that it is possible to control the structure and thickness of the electroactive biofilm on the anode by adjusting the shear force (e.g., flow rate of the pump) [33]. This technique is applied for the GDE to remove the unwanted bacteria from the surface of the GDE.
(5) The functional amino groups are known for their antibacterial effect. The negatively charged cell membranes adsorb to the positively charged amino groups, resulting in damage to the cell. Therefore, a coating with these groups might prevent bacterial growth of the GDE [34].

For the first time, five possible anti-fouling strategies were investigated and compared with a new reactor concept based on rotating cathodes.

## 2. Materials and Methods

### 2.1. Electrodes

Gas diffusion electrodes were prepared based on a stainless-steel mesh as conductive current collector, a gas diffusion layer and catalyst layer, respectively. The catalysts used are Printex 6L carbon (Orion Engineered Carbons S.A., Houston, TX, USA), MnO_2_ (EMD Millipore Corporation, Burlington, MA, USA) and MoS_2_ (Metallpulver24 Corp., Sankt Augustin, Germany) in a ratio of 30:2:1. This catalyst mixture was dispersed with a PTFE dispersion (TF 5135GZ, Dyneon GmbH, Neuss, Germany), a methylcellulose solution (1 wt.% Walocel MKX 70,000 PP01, Dow Deutschland Anlagengesellschaft mbH, Bomblitz, Germany), isopropanol and deionized water using an ultrasonic sonotrode (Hielscher Ultrasonics GmbH, Teltow, Germany). For all electrodes, the ratio of catalyst to PTFE was 6:4. The resulting suspension was applied to a previously degreased stainless-steel mesh with a mesh size of 100 µm × 100 µm and a wire diameter of 65 µm (Spörl KG Präzisionsdrahtweberei, Sigmaringendorf, Germany). An automatic spraying machine spread the coating evenly to both sides of the mesh using an ultrasonic nozzle (Hielscher Ultrasonics GmbH, Teltow, Germany). After the spraying process, the electrodes were pressed at 74 kg cm^−2^ at a temperature of 130 °C and afterwards sintered at temperatures up to 330 °C. All of the manufactured GDEs have an active area of approx. 204.2 cm^2^ and a loading of 9.0 ± 0.2 mg cm^−2^.

Anode plates were made of a compound material with 86% graphite and 14% polypropylene as binder, called PPG86 (Eisenhuth GmbH & Co. KG, Osterode am Harz, Germany). The surfaces of the anode plates were roughened by a wire brush to enlarge the surface and to provide a better accumulation of the exoelectrogenic bacteria. The total area of the anode plates is 277.65 cm^2^.

### 2.2. Reactor Design and Components

All experiments were carried out with a reactor with its rotating cathode as centerpiece. For each reactor, two cathodes were mounted to a PVC supporting element. This element was glued onto a hollow shaft with an outer diameter of 32 mm (also PVC). Two holes (opposite) were drilled from the supporting element to the hollow shaft. A flexible tube was glued to one hole going to one end of the hollow shaft, ending in a plug with another hole. This was necessary to pump air through the hollow shaft directly into the supporting element to provide enough air for the GDEs. The second hole in the hollow shaft functions both as an air outlet and for the cable gland. A common copper cable was used to connect the GDEs. Therefore, the cable was soldered to a thin copper foil. When mounting the GDEs to the supporting element, the GDEs were pressed right onto the copper foil by screwing an annular fixing element. A sealing with the same shape as the annular fixing element was placed between GDEs and supporting element.

The supporting element was manufactured inversely to provide two GDEs on each side of the supporting element.

A hollow shaft with the supporting element and mounted GDEs was positioned in a reactor tank. This tank was also made of PVC. Commercially available radial shaft seals and axle bearings were used to keep the hollow shaft in place. A commercially available 24 V electric gear engine (SWF 405923 GMPD, Wald-Antriebe, Walsrode, Germany) was used to rotate the shaft. The rotation of the shaft was adjusted to approx. 20 min^−1^ by potentiometers (KEMO M195 PWM, Voelkner, Nürnberg, Germany).

The two GDEs in each reactor were connected to the in-house air pressure system and the air flow was adjusted to approx. 10 L/min, which is hyperstoichiometric.

After installing the two GDEs, anode plates were integrated into the tank. They were held in place by a rack and connected by cable shoes. The number of anode plates depends on the reactor configuration, see Table 2.

The inner dimensions of the tank are ca. 30 cm (height), ca. 30 cm (width) and ca. 30 cm (depth). The tank has an overflow. After assembling all components to the reactor, the reactor was filled until the overflow was reached (ca. 17 L wastewater). The reactor tank was covered with a transparent lid to avoid air entry.

All reactors were heated to about a constant temperature of 25 °C by an aquarium heater (Aquael Ultra Heater, 100 W, Naqua, Schönaich, Germany) for faster biofilm growth and for better comparability between different reactors.

**Figure 1:**
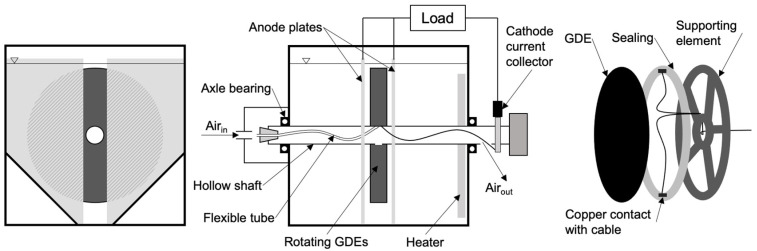
Schematic of reactor design.

A schematic of the reactor design is shown in Figure 1. The left figure is a cross section of the reactor with focus on the electrodes’ geometry. The middle figure (90° turn) illustrates the configuration of reactor components, and the right figure is an exploded view of the GDE, sealing and supporting element.

The active area of the anode plates (82.23 cm^2^, see shaded area in Figure 1) is smaller than the active area of the GDEs, determined by the overlapping of GDE and anode. The power densities (PD) were calculated by dividing the absolute power of the reactor by the active cathode area, which is covered with electrolyte (see Table 2), following Equation (1).

PD = U × I × A_cat_^−1^ (1)

**Figure 2:**
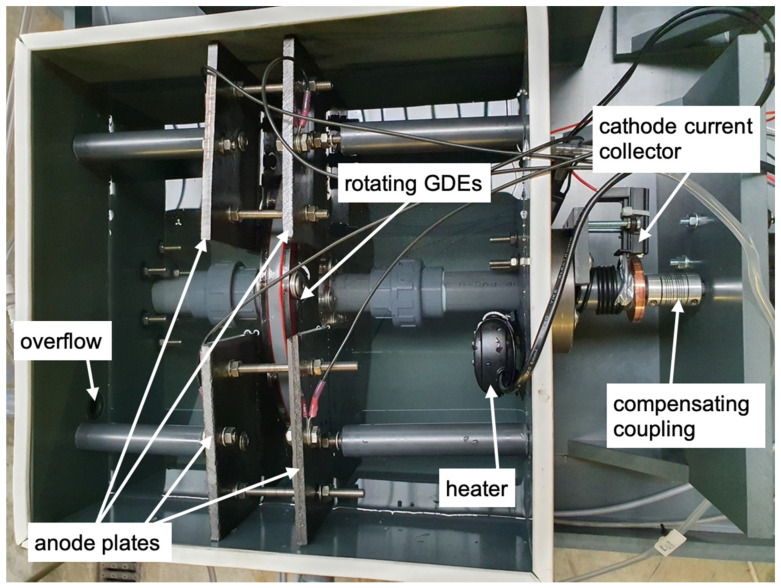
Reactor set-up, filled with tap water for tightness testing (view from top).

Figure 2 shows a photo of the reactor detailly described in Figure 1 from the top view. The main components are marked in the picture.

All process parameters (current, voltage, rotation speed, temperature, etc.) were controlled, adjusted and recorded by a process control system (National Instruments Labview). A constant current source (CCS) was connected to anode and cathode to control the current between both electrodes [35].

Additionally, a Raspberry Pi camera (HQ cam) was mounted into one reactor to visualize the biofilm growth. The Raspberry Pi stopped the rotation of the cathode element, then turned on an LED light (cool white) and took the picture. Then the rotation of the cathode element was turned on again. A photo was taken every three hours.

### 2.3. Anti-Fouling Strategies

In this work, five anti-fouling strategies were investigated. All anti-fouling techniques are already mentioned in the introduction. For use in an MFC, these techniques have to be adapted.

(1) An UV-LED (Stanley ULM1B, Moerfelden-Walldorf, Germany) was used in one reactor for the killing of microbials of the surface of one GDE. This LED has a spectrum of 259–269 nm wavelength, is commercially available, has low power consumption and a small size (32 mm in diameter, 17 mm in depth), so that it fits into the reactor design. According to the technical data of the UV-LED, the irradiance is in the range of 50 to 70 µW cm^−2^ within a square of 5 cm × 5 cm with a 10 cm distance to target. The LED was mounted onto a thin plate of stainless steel and fixed between two anode plates. The distance between LED and GDE surface is approx. 2 cm.
(2) In another reactor, the use of two electrodes with an additional copper compound coating was investigated. For this purpose, copper resinate was dissolved in acetone and the solution was subsequently filtered. The solutions prepared had concentrations of 0.86 wt.% and 1.72 wt.%. Afterwards, the gas diffusion electrodes were brushed one-sided with one of the two solutions. For reasons of comparison, the coating was only applied to one half of the electrode. The other half remained untreated.
(3) Polarity reversal is used in conventional electrolysis cells to remove scaling (inorganic compounds) on the cathode. The operation procedure had to be adapted to the reactor design in this work. In normal operation mode, anode and cathode are connected for electrical power generation. During polarity reversal, a contactor switches the anode currentless, while the cathode is connected to an auxiliary anode (made of stainless-steel mesh). An external power source (Peaktech 6095, Bürklin, Oberhaching, Germany) supplies the cathode and the auxiliary anode with approx. 8 V and 0.1 A to start electrolysis. Consequently, oxygen is produced at the auxiliary anode, while hydrogen peroxide is produced on the cathode [36]. Hydrogen peroxide is widely known and used as disinfectant [37].
(4) A simple technique to remove most of the biofilm is mechanical abrasion by knifes (see [17]) or wipers. As mentioned above, this technique is not suitable for the in-house production GDEs, because this procedure would destroy the GDE. For that reason, another mechanical method was investigated. The GDE is mounted onto a rotating shaft. It is possible to adjust the rotation speed of the shaft to obtain higher flow velocities of the electrolyte to remove the biofilm by shear stress. The maximum rotation speed of the shaft is limited by the electric gear engine, which is 50 min^−1^. For the experiments, the rotation speed of the shaft was set to the maximum for one minute every hour.
(5) Electrode-membrane units were fabricated to investigate the effect of an antibacterial membrane on biofilm formation. For this purpose, a membrane solution was prepared, consisting of 5% Fumion^®^ FFFA-3 shredded film (FUMATECH BWT GmbH, Bietigheim-Bissingen, Germany) in a mixture of propanol and butanol in a ratio of 1:1. The membrane solution was applied to one side of the gas diffusion electrode using a brush. Immediately afterwards, the antibacterial membrane (fumasep^®^ FFFA-3–30, FUMATECH BWT GmbH, Bietigheim-Bissingen, Germany) with a thickness of 30 µm was applied to the electrode. The membrane solution acts as an adhesive, so that after evaporation of the solvent a bonded membrane-electrode-assembly (MEA) is formed. The type of membrane is an anion exchange membrane with an ion exchange capacity of 1.66–2.04 mmol g^−1^ at thickness of 10 µm. Two of these manufactured MEAs were installed in an MFC for examination.

In general, all reactors have the same set-up for comparability, but small varieties were unavoidable. Reactor 3 housed only two anode plates, as the other two anodes were replaced by stainless steel meshes. Reactor 1 was only half full with wastewater because the light rays of the UV-LED should pass through air and not wastewater, otherwise the rays would be weakened and this anti-fouling method would be ineffective. Table 2 shows the configuration of each reactor.

### 2.4. Electrolyte

All reactors were filled with real wastewater from a domestic wastewater treatment plant which originally contains electroactive bacteria. Wastewater was taken from the effluent of the primary clarifier to minimize the amount of inorganic solids in the reactor tank. The concentrations of the main wastewater components after the primary clarifier are shown in Table 3.

Each reactor was connected to a peristaltic pump (Dosatronic DOSAFlex, Ravensburg, Germany) for an automatic spiking process to keep the chemical oxygen demand between 200–300 mg L^−1^. The spiking substrate was a solution of 0.5 wt.% sodium acetate waterfree (CAS 127-09-3, Th. Geyer, Höxter, Germany), 0.5 wt.% d-glucose (CAS 50-99-7, Th. Geyer, Höxter, Germany) and tap water. Every three hours, 45 mL (18 mL for the reactor with UV-LED) of the substrate was pumped into each reactor. The rotation of the shaft with its GDEs ensures a proper dispersion of the substrate in the reactor volume.

## 3. Results and Discussion

**Figure 3:**
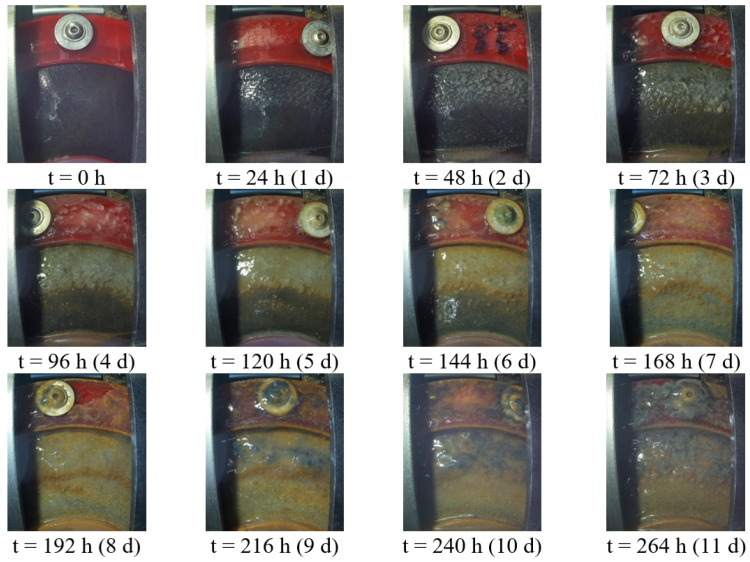
Biofilm development on an untreated cathode during 11 days.

Figure 3 shows the biofilm growth during 11 days in an MFC without anti-fouling strategy, taken by the Raspberry Pi camera.

Within 24 h after filling the reactor with wastewater, a biofilm on the surface is visible. After seven days, the complete surface is covered with a biofilm. In the following days, the biofilm grows to a thickness of approx. 3 to 5 mm, manually measured after disassembling the GDE. Then the biofilm was removed from the GDE for organic dry matter analysis and qualitative bacteria screening by Amodia Bioservice GmbH. The average organic content of the samples, based on the dry matter, is about 80.3%. DNA sequence analysis confirmed the presence of acidaminococcus fermentans, victivallis lenta, acidaminococcus intestini and parabacteroides chartae. These are all anaerobic and mesophilic bacteria.

**Figure 4:**
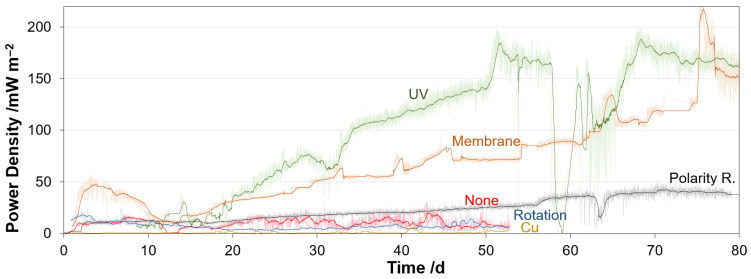
Development of power densities over time within 80 days for the anti-fouling strategies UV-irradiation, membrane addition, polarity reversal, rotation speed and copper layer addition compared to untreated GDE.

The reduction of the power generation of the cells does not correlate with the biofilm growth in the beginning (see Figure 4). The biofilm seems not to affect the power generation initially, as differences in the performance of the reactors with and without antifouling strategy become evident only from day 15 onwards. This is likely because the biofilm at the beginning is thin enough so that the ions can still migrate through the biofilm in a sufficient number. The electrical current between the anode and cathode, and thus the ion exchange, is very low compared to chemical fuel cells.

It is also likely that the biofilm grows inside the pores of the GDE, so that the bacteria may use the oxygen of the GDE.

**Figure 5:**
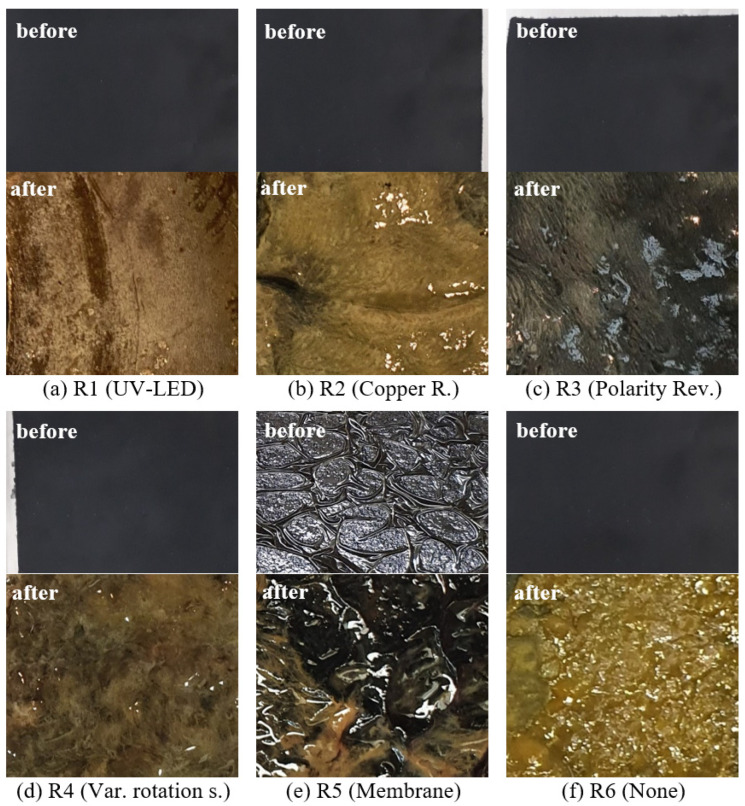
Photos of biofilms on GDEs after disassembling.

Within 80 days, the power densities of the five reactors show different developments. The lowest power density (PD) was obtained with Reactor 2 (copper resinate). This low PD could be explained by the coating itself. The resinate formed a compact layer on the GDE surface, which might have closed all the GDE’s pores, which reduced the performance of the GDE drastically. A visual inspection of the GDE shows complete biofilm growth (Figure 5b). It is possible that the copper in the resinate is not accessible and could not develop its antibacterial effect.

The second lowest PD was achieved with Reactor 4 (varying rotation speed). It is obvious that the highest rotation speed of the GDE element was not high enough to prevent biofilm growing on the GDE surface. A higher rotation speed might be more sufficient in biofilm preventing, but a higher rotation speed could not be adjusted due to the motor parameters. The achieved PD with that reactor stayed almost constant over the time with a maximum of approx. 20 mW m^−2^ at the beginning. An examination of the GDE confirms the assumption. The entire electrode surface is affected by a biofilm (Figure 5d).

Reactor 3 (polarity reversal) obtained quite better PDs, rising to a maximum of approx. 45 mW m^−2^ at day 70. The polarity reversal seems to be a potential strategy for biofilm inhibition on the GDE surface, as it has a positive effect on the PD. This can also be verified by the surface of the GDE, which shows less biofilm growth than an untreated electrode (Figure 5f). The thickness of the microbial film is quite thin so that the black electrode surface underneath is still visible (Figure 5c).

The highest PDs were achieved with Reactors 1 (UV) and 5 (membrane). Both reactors reached a maximum PD of about 200 mW m^−2^, but after 75 days both reactors reached a stable PD of approx. 150 mW m^−2^. Both reactors showed an increasing power generation, but Reactor 1 benefitted from a faster increasing PD. The PD of the reactor with the UV-LED decreased at day 58, because the feed was interrupted.

Accordingly, the use of the antibacterial membrane and the UV irradiation seems to reduce the biofilm infestation. This can be confirmed by the photos of the removed cathodes. The irradiated electrode does not show any biofilm on its surface. Only scaling effects can be seen (Figure 5a). The GDE coated with a membrane shows slight biofilm growth but the black surface of the electrode can still predominantly be seen (Figure 5e). In addition, the biofilm does not appear to be cross-linked to the membrane, allowing an easy removement.

## 4. Conclusions and Outlook

In this contribution, a new MFC reactor concept was investigated and successfully operated. Five different anti-fouling strategies to prevent biofilm growing on GDEs were integrated in this new reactor design and compared to a GDE without any anti-fouling strategy. All strategies have in common that they work in situ, which means that the GDE did not have to be removed from the reactor.

Comparing the power generation and the biofilm growth on the electrode, UV and membrane were the most promising anti-fouling strategies. Although the power generation of the reactor with UV-LED was much higher in the beginning, membrane as anti-fouling strategy is recommended. This is mostly due to the high operational costs. The UV-LED works at approx. 6.1 V and 0.25 A. The consumption will be 36.6 Wh d^−1^, if the UV-LED is operated continuously. This means the UV-LED consumes more power than the MFC produces. For future application, the duration of the UV irradiation should be reduced to enable net power generation by MFC. Additionally, the use of UV-LEDs in stack systems is another challenge, as there must be a UV-LED on each side of the rotating cathode.

Coating the GDE with a membrane causes some higher production costs, but no additional energy consumption during operation.

The membrane also causes a better separation of the cathode chamber from the anode chamber and prevents oxygen crossover from the GDE backside to the wastewater. For future application, the membrane should be prepared by coating the GDE with a sprayable solution of the membrane material. This approach avoids the gluing of an additional membrane and simplifies the production of MEAs.

The in situ prevention of biofilm growth on chemical catalytic GDEs is appreciated as a pre-requisite towards technical scale wastewater treatment applications of single-chamber MFC using GDEs, because a long-term, low-cost operation is necessary for commercial breakthrough of MFCs. Costs for antifouling strategies, e.g., for energy (polarity reversal, UV-LED) as well as for maintenance (e.g., cathode replacement) need to be minimized. Antifouling membrane coating seems to be a promising technology for the desired application, as they may prevent biofilm growth and guarantee long-term operation of MFC with sufficient power output.

## Figures and Tables

**Table 1:** Selected single-chamber microbial fuel cells with antifouling (AF) investigations.

Cathode (Area/cm^2^)	Anode	Medium	Max. Power Density /mW m^−2^	AF Strategy	In Situ	Period /d	Volume /mL	Lit.
AC ^1^ GDE (6.2)	Graphite fiber brush	Domestic wastewater	132 ± 7	Scraping with a magnet	Yes	30	28	[10]
GDE (15.9)	Carbon cloth	Inoculated with compost leachate + acetate Medium: synthetic	1960	Reactor design with easily replaceable electrodes	No	25	1800	[11]
C GDE (24.18)	Carbon fiber veil	Inoculated with activated sludge from existing MFCs Feed: fresh human urine	44 ± 13 *	Washing with NaOH and lysis solution, removing the external layer and re-painting	Yes	90	11.4	[12]
AC ^1^ GDE (7)	Carbon fiber brush	Inoculated with effluent from existing MFCs electrolyte: PBS ^2^ + acetate	1842 ± 80	Half-wave rectified alternating fields	-	20–30	28	[13]
C GDE (7)	Carbon fiber brush	Feed: 20% domestic wastewater, 80% glucose + PBS ^2^	892 ± 8	Heating and Ultrasonic concussion	No	180	28	[14]
Ag/FeS/PGC ^3^ GDE (7)	Carbon fiber brush	Inoculated with effluent from existing MFCs electrolyte: PBS ^2^ + glucose	1361	Ag/FeS/PGC ^3^ catalysts	Yes	90	28	[15]
AC ^1^ GDE + Enrofloxacin (7)	Carbon fiber brush	Inoculated with effluent from existing MFCs + acetate + PBS^2^	1069.7 ± 10.5	Enrofloxacin	Yes	91	28	[16]
AC ^1^ GDE (19.6)	Carbon felt	Inoculated with effluent from existing MFCs Feed: artificial growth medium	1800	Removing by a paper knife, Washing with NaOH or HCl, Drying and Repressing **	No	365	60	[17]
AC ^1^ GDE (7)	Carbon fiber brush	Inoculated by the effluent of a laboratory MFC Feed: Acetate + PBS ^2^	1040.1 ± 35.7	Cleaning with SDS and NaOH	No	180	28	[18]
AC ^1^ GDE (12.6) *	Carbon fiber brush	Inoculated with effluent from existing MFCs + Glucose + PBS ^2^	1171 ± 71	Varying hydrophobicity by different proportions of PTFE and LA132	Yes	150	28	[19]
C GDE + Ag_3_Pt (4.9) *	Carbon felt	Inoculated with treated septic tank mix consortia Substrate: fish market wastewater	999 ± 31	Ag_3_Pt catalyst	Yes	80	14.7	[20]
AC ^1^ GDE + QAC ^4^ (?)	Carbon fiber brush	Inoculated with effluent from existing MFCs Medium: NaCl, acetate	1041 ± 12	Bifunctional QAC ^4^	Yes	60	28	[21]
AC ^1^ GDE + Fe/CB ^5^ (7)	Graphite fiber brush	Inoculated with effluent from existing MFCs Medium: acetate, PBS ^2^	1410 ± 50	Cleaning with HCl, removing biofilm gently	No	510	28	[22]
CB ^5^ GDE (4 × 180)	SSM ^6^	Inoculated with heat treated anerobic sludge Feed: acetate + NaHCO_3_ + NH_4_Cl + CaCl_2_.2H_2_O + MgSO_4_.7H_2_O + K_2_HPO_4_ + KH_2_PO_4_ + Trace metals	275 mW/m^3^	Varying hydrophobicity with PVA and PTFE Using biocide vanillin	Yes	50	4 × 300	[23]
C GDE + AgNP ^7^ (300)	SSM ^6^	Inoculated with treated septic tank mix consortia Medium: fish market wastewater	9.8 ± 0.3 W/m^3^	Ag-NPs ^7^ catalyst	Yes	36	230	[24]
C GDE + MnO_2_ + MoS_2_ (2 × 204.2)	SSM ^6^	Inoculated with domestic wastewater Feed: acetate + glucose	200	UV radiation Cu Coating Polarity reversal Varying rotation speed Antibacterial membrane	Yes	53/80	17,000	This paper

^1^ activated carbon; ^2^ phosphate-buffered saline; ^3^ partly-graphitized carbon; ^4^ quaternary ammonium compound; ^5^ carbon black; ^6^ stainless steel mesh; ^7^ nano particles; * calculated from data; ** some of the electrodes were additionally prepared using one-year old AC and new PTFE gas diffusion membrane.

**Table 2:** Configuration of the investigated reactors.

Number.	1	2	3	4	5	6
Anti-fouling method	UV-LED	Coating with copper resinate	Polarity reversal	Varying rotation speed	membrane	None (for comparison)
Anode	4 × PPG86	4 × PPG86	2 × PPG86	4 × PPG86	4 × PPG86	4 × PPG86
Active cathode area	0.0204 m^2^	0.0408 m^2^	0.0408 m^2^	0.0408 m^2^	0.0408 m^2^	0.0408 m^2^
Cathode	in-house GDE	in-house GDE	in-house GDE	in-house GDE	in-house GDE	in-house GDE
Reactor filling level	half-full	full	full	full	full	full

**Table 3:** Composition of wastewater after primary clarifier (for year 2021, approx. values).

	COD mg L^−1^	NH_4_^+^ mg L^−1^	NO_3_^−^ mg L^−1^	P_total_ mg L^−1^
Min. Max.	82.5 953.5	11.0 57.9	<1 <1	2.8 11.4
Average	334.7	31.2	<1	6.4

## Data Availability

Not applicable.
